# Mobile laminar air flow screen for additional operating room ventilation: reduction of intraoperative bacterial contamination during total knee arthroplasty

**DOI:** 10.1007/s10195-011-0168-5

**Published:** 2011-11-10

**Authors:** D. Sossai, G. Dagnino, F. Sanguineti, F. Franchin

**Affiliations:** 1 Health Safety and Prevention Department, A.O.U. San Martino, Genoa, Italy; 2 Italian Institute of Technology (IIT), Genoa, Italy; 3 Clinica Ortopedica Università, A.O.U. San Martino, Genoa, Italy

**Keywords:** Total knee arthroplasty, Laminar air flow, Infection

## Abstract

**Background:**

Surgical site infections are important complications in orthopedic surgery. A mobile laminar air flow (LAF) screen could represent a useful addition to an operating room (OR) with conventional turbulent air ventilation (12.5 air changes/h), as it could decrease the bacterial count near the operating field. The purpose of this study was to evaluate LAF efficacy at reducing bacterial contamination in the surgical area during 34 total knee arthroplasties (TKAs).

**Materials and methods:**

The additional unit was used in 17 operations; the LAF was positioned beside the operating table between two of the surgeons, with the air flow directed towards the surgical area (wound). The whole team wore conventional OR clothing and the correct hygiene procedures and rituals were used. Bacterial air contamination (CFU/m^3^) was evaluated in the wound area in 17 operations with the LAF unit and 17 without the LAF unit.

**Results:**

The LAF unit reduced the mean bacterial count in the wound area from 23.5 CFU/m^3^ without the LAF to 3.5 CFU/m^3^ with the LAF (*P* < 0.0001), which is below the suggested limit for an OR with ultraclean laminar ventilation. There were no significant differences in the mean bacterial count in the instrument table area: 28.6 CFU/m^3^ were recorded with the LAF (*N* = 6) unit and 30.8 CFU/m^3^ (*N* = 6) without the LAF unit (*P* = 0.631). During six operations with LAF and six without LAF, particle counts were performed and the number of 0.5 μm particles was analyzed. The particle counts decreased significantly when the LAF unit was used (*P* = 0.003).

**Conclusion:**

When a mobile LAF unit was added to the standard OR ventilation, bacterial contamination of the wound area significantly decreased to below the accepted level for an ultraclean OR, preventing SSI infections.

## Introduction

Surgical site infections (SSI) represent one of the most common complications in surgery. In particular, deep periprosthetic infections in orthopedic surgery constitute a disaster for both patient and surgeon.

Conservative estimates of infection rates average 1–2% for hip implants and 2–4% for knee implants [1, 2]. The number of joint replacements is expected to double in the next 20 years, and if the infection rate is not reduced, the incidence of infection will also double, yielding increased morbidity, hospital stays, and costs for the healthcare system [3, 4].

Periprosthetic infection rates have been shown to correlate with the number of airborne bacteria within 30 cm of the wound [5]. This is influenced by several factors relating to either the surgical environment (number of operating theater personnel, their clothing, type of ventilation system used) or the surgical procedure employed (approach, duration of exposure, use of a tourniquet).

The source of pathogens can be the patient (endogenous infection), the bacteria present in the OR air, instruments used, or the surgeon’s hands (exogenous infection). However, it is generally accepted that the main cause of surgical site infections (SSIs) after clean operations is bacterial contamination of the OR air, predominantly from contaminated skin scales shed by the surgical team, instruments used, or the surgeon’s hands [6–8].

Small numbers of organisms, including those of low pathogenicity, can cause orthopedic implant infections, and give rise to a considerable degree of morbidity and also mortality. It has been estimated that as few as ten colony forming units (CFU) are sufficient to cause deep infection in a prosthetic replacement arthroplasty. Bacteria that cause infection in the joint after total hip or knee replacement are inoculated into the wound at the time of insertion of the prosthesis [6, 9, 10].

The number of airborne bacteria in the OR is also dependent on the number of people present as well as their activities and behavior. Use of a ventilation system and appropriate personnel dress and discipline are therefore ways to reduce air contamination in the OR [1, 2, 4, 7–9, 11–26].

A laminar air flow ventilation system is recommended for an OR where orthopedic implant surgery is performed [9, 11]. Unfortunately, LAF systems are very expensive and complicated to install in a pre-existing OR. The introduction of a mobile LAF screen to complement the use of a standard ventilation system could be an effective but inexpensive way to decrease bacterial air contamination, as noted by Friberg et al. [7, 12] and Pasquarella et al. [8].

The aim of this study was to evaluate the efficacy of LAF at reducing bacterial contamination in the surgical area during orthopedic implant surgery in an OR with conventional turbulent air ventilation.

## Materials and methods

### Surgery

This study focused on 34 total knee replacements carried out over a period of two months.

All operations were performed in the same OR, early in the morning, and by the same surgeon; all cases received spinal anesthesia, tourniquet, and standard short-term antibiotic prophylaxis.

The additional LAF screen was used in 17 operations, while the remaining 17 operations were performed under ordinary conditions without the additional LAF screen.

### Operating room

The experiments were performed within a standard OR (≈120 m^3^) equipped with a conventional turbulent ventilation system (with 12.5 air changes/h).

**Fig. 1:**
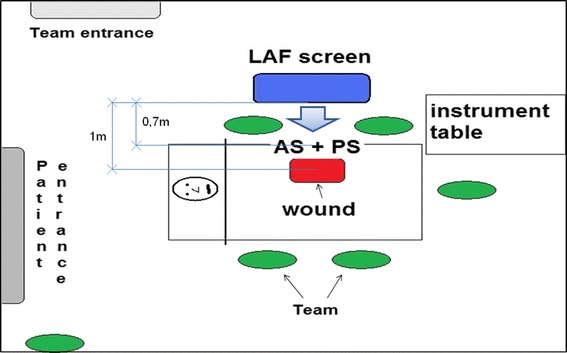
Schematic setup used during the experiments; *AS* air sampler (bacteria), *PS* particle sampler. AS and PS were located 30 cm from the wound

Mean thermohygrometric parameters were [11]: temperature, 20.6°C (0.1); relative humidity, 44.6% (3.1) (Table 1; Fig. 1).

**Table 1:** OR data: relative humidity and temperature are expressed as mean (standard deviation)

Operating room data	
Volume	120 m^3^
Ventilation system	Turbulent
Air changes	12.5 v/h
Relative humidity	44.6% (3.1)
Temperature	20.6°C (0.1)

### Additional LAF screen

The additional LAF screen used in the study (Toul-400, Toul Meditech, Vasteras, Sweden) is a mobile unit that produces ultraclean exponential laminar air flow in a predefined area (the wound in our case). The additional mobile unit is a box with a fan and a HEPA filter (CAMFIL type, 99.997% particles >0.3 μm). The screen produces a laminar air flow of 0.5–0.7 m/s onto the wound and 0.4 m/s at the periphery. This exponential air flow prevents the entrainment of OR air outside the LAF [7, 8]. This air flow is turbulence-free and not impeded by the movements of the surgical team in the defined air flow area. The existing regular ventilation system does not affect the functioning of the unit. A camera assists in determining the direction of air flow, and an integrated sensor determines the correct distance from the surgical site for maximum effect. An integrated display allows the user to easily check and verify the setup (Toul Meditech data).

The LAF screen was positioned beside the operating table and between the two surgeons, with the air flow directed towards the surgical area, as shown in Fig. 1.

### Surgical team

The surgical team consisted of a chief surgeon, an assistant surgeon, and two residents; there were also one chief anesthetist, one resident, one scrub nurse, one room nurse, and a technician. The team numbered between six and eight during all 34 operations.

Each member of the team wore conventional OR clothing during all operations: the surgeons, scrub nurse, and technician wore sterile nonwoven gowns, facemasks, surgical caps, and sterile gloves; the anesthetist and room nurse wore a woven OR uniform (shirt and trousers), facemask, surgical cap, and nonsterile gloves.

### Sampling methods

Air contamination (in CFU/m^3^) was studied during all operations using a Biotest (Rockaway, NJ, USA) RCS Plus sampler (50 l/min) using Biotest HYCON agar strips TC-γ (γ-irradiated Total Count Tryptic Soy agar). The sampler was located 30 cm from the wound. Air counts were performed during 17 operations with the LAF screen and 17 operations without the LAF. Air quality on the instrument table was also investigated in six operations with and without the LAF unit. Sampling periods were always 20 min, and 1 m^3^ of air was sampled, as suggested by ISPESL guidelines [11] (Fig. 1; Table 2). The agar strips were incubated for 48 h at 37°C before counting the CFU, and the results were expressed in CFU/m^3^ (Table 3).

**Table 2:** Sampling methods used in the experiment

	Counts of bacteria in the air (UFC/m^3^, using a Biotest RCS sampler)	Counts of particles in the air (particles/m^3^, using a Biotest APC sampler)
Sampling capacity	50 l/min	2.8 l/min
Sampling time	20 min	20 min
Sample volume	1 m^3^	56 l
Sampling locations in wound area	34 (17 with LAF and 17 without LAF)	12 (6 with LAF and 6 without LAF)
Sampling locations in instruments table area	12 (6 with LAF and 6 without LAF)	12 (6 with LAF and 6 without LAF)

**Table 3:** Bacterial air contamination (CFU/m^3^) in the wound area and the instrument area with and without an additional LAF screen; mean (standard deviation) values and significant differences are indicated in bold

Bacterial contamination (CFU/m^3^ of the:	With LAF	*P* value	Without LAF
Wound (CFU/m^3^)	*N* = 17		*N* = 17
	**3.5** (2.4)	<0.0001	**23.5** (9.6)
Instrument table (CFU/m^3^)	*N* = 6		*N* = 6
	**28.6** (15.2)	ns (0.631)	**30.8** (15.3)

Particle counts were performed in the wound area during 12 operations (six with the LAF screen and six without) at the same time as the bacteria count using the Biotest (Dreieich, Germany) APC Plus (2.8 l/min). The sampling periods were again 20 min (Fig. 1; Table 2). Results were expressed in particles/m^3^, and ISO values for 0.5 μm particles were considered when interpretating the results [14] (Table 4).

**Table 4:** Particle counts (0.5 μm) in the wound area and instrument area with and without the additional LAF screen; mean (standard deviation) values and significant differences are shown in bold

Particle count (0.5 μm/m^3^) of the:	With LAF	*P* value	Without LAF
Wound (0.5 μm/m^3^)	*N* = 6		*N* = 6
	**17,361** (22,634)	0.003	**970,533** (576,286)
Instrument table (0.5 μm/m^3^)	*N* = 6		*N* = 6
	**1,224,367** (723,796)	ns (0.521)	**1,380,181** (791,851)

### Statistical analysis

SPSS (Statistical Package for Social Sciences) was used for statistical evaluations. The Mann–Whitney *U* test was used to establish significant differences between means. *P* ≤ 0.02 was regarded as significant.

## Results

Mean bacterial air contamination in the wound area was 23.5 CFU/m^3^ under standard ventilation conditions; when the LAF unit was added to the standard ventilation, the mean bacterial count in the wound area decreased to 3.5 CFU/m^3^ (*P* < 0.0001) (Table 2), a reduction of about 85%, which is below the accepted limit (<10 CFU/m^3^) for ultraclean laminar ventilation [6, 9, 11, 13, 15].

In the instrument table area, the mean bacterial air contamination was almost the same whether or not the LAF unit was used (*P* = 0.631 = ns): 28.6 CFU/m^3^ with the LAF unit and 30.8 CFU/m^3^ without the LAF (Table 3).

When the LAF unit was used, there was a significant correlation between bacterial air contamination on the wound and instrument table (*P* = 0.0004); without the screen, no significant correlation was found (*P* = 0.361).

The mean numbers of 0.5 μm particles in the wound area and the instrument table area are shown in Table 4. Without the LAF screen, the mean value in the wound area was 970,533 particles/m^3^; upon adding the LAF screen, this mean was reduced to 17,361 particles/m^3^ (*P* = 0.003).

In the instrumental table area, the mean particles/m^3^ value ranged from 1,224,367 with the LAF unit to 1,380,181 without the LAF unit (*P* = 0.521 = ns).

No significant correlation was found between the particles/m^3^ values in the wound area and the instrument table area when the LAF unit was not used (*P* = 0.262); but when the LAF unit was added, a significant correlation was observed (*P* = 0.004).

## Discussion

Many studies have demonstrated a correlation between airborne bacterial contamination and postoperative joint sepsis in arthroplasty surgery [5, 6, 9, 13, 22]. Correct rituals, surgical clothing, and the use of ultraclean laminar ventilation are strongly recommended in orthopedic implant surgery [7–9, 11–13, 18, 20–22] in order to reduce postoperative SSIs and respect the accepted limit of 10 CFU/m^3^ in the wound area for an ultraclean OR [14, 15].

We tested the efficacy of using an LAF unit in addition to a conventional air ventilation system in an implant surgery OR (12.5 air changes/h). We studied the effect of the screen on bacterial OR contamination during 34 total knee replacement operations. During the experiments, the surgical team respected the OR rituals and hygiene procedures and wore proper surgerical clothing. In our study, we used an active sampling method, as suggested in the ISPESL guidelines [11]. Bacterial sampling was performed in the wound area and the instrument table area to get an indication of bacterial contamination levels present under standard ventilation conditions and when the LAF screen is also used. We also decided to perform particle sampling in the same places and at the same time as the bacterial sampling during 12 operations (six with LAF and six without LAF), as an additional indicator of LAF unit efficacy.

The results suggested that the LAF screen is very effective at reducing bacterial contamination; the CFU/m^3^ value in the wound area was below the accepted limit for an ultraclean OR: the contamination in the wound area dropped from 23.5 CFU/m^3^ under standard ventilation conditions to 3.5 UFC/m^3^ when LAF was used, which is well below the limit of 10 CFU/m^3^ accepted for ultraclean laminar ventilation [6, 9, 13] and that of the UK (NHS) standard HTM 2025, which states that a limit of 20 CFU/m^3^ should not be exceeded in an OR with ultraclean laminar ventilation during surgical operations [11, 15]. This reduction in the CFU/m^3^ value in the wound area is statistically significant.

Bacterial contamination of the instrument table area did not change upon adding the LAF screen: it was 28.6 CFU/m^3^ with the LAF unit and 30.8 CFU/m^3^ without the LAF unit; no significant correlation between the level of contamination and whether LAF was functioning was observed, meaning that the influence of the LAF unit was limited to the focal area (in this case the wound area). The count of 0.5 μm particles in the wound area dropped from 970,533 particles/m^3^ when LAF was added to 17,361 particles/m^3^ when LAF was not working. This means that, for the wound area, the OR complied with ISO Class 8 standard conditions, and with ISO Class 6 standard conditions when the LAF unit was used. In the instrument table area, the particles count complied with ISO Class 8 conditions [14].

In conclusion, the prevention of infection is preferable to treatment in terms of both patient outcome and cost of treatment [4, 23–25]. Employing an additional ultraclean LAF unit reduced bacterial contamination and bacteria-carrying airborne particles in the surgical area (the wound) during total knee replacement operations. The CFU/m^3^ value in the wound area was reduced to below the limit suggested for implant surgery performed in an OR with ultraclean laminar ventilation.

A complete ultraclean LAF ventilation system is very expensive, and can sometimes be impossible to install in pre-existing premises without extensive rebuilding [3, 7]. Employing an additional LAF screen could be an interesting, effective, and inexpensive complement to OR standard ventilation when laminar air flow is required; in other words, for high-risk surgery (implant surgery, neurosurgery, transplant surgery), and in situations with insufficient ventilation or clothing facilities to reduce bacterial contamination and prevent SSI infections [7].
